# The Wassermann Reaction

**Published:** 1909-08

**Authors:** 


THE WASSERMANN REACTION.—Since Wassermann's announcement in 1906 of his blood test for syphilis much work of a confirmatory nature has been done by independent investigators in the serodiagnosis of this disease. The principle upon which the reaction was based owes its inception to the researches of Bordet and Gengou. Wassermann arrived at the conclusion that the sera of syphilitics contained substances (not present in the sera of non-syphilitics) which have "an avidity for certain substances contained in luetic and normal organs, and also in guinea pigs, and for certain lipoid bodies, as lecithin, cholestrin, etc." (Butler, *J. A. M. A.*, September 5, 1908.)

Elaborating and developing this idea, he formulated the Wassermann reaction, which he regards as practically specific in the diagnosis of all forms of syphilis.

Owing to the complicated principles involved and to the complexity of the method employed by the discoverer, the test will for some time be possible only in well-equipped laboratories and by more or less expert workers. The ordinary physician, to whom so important a method of diagnosis must appeal especially, will find himself compelled to either turn his case over to the laboratory man or await the evolvement of some simpler means of making the test. That this much-to-be-desired consummation is not far distant may be inferred from the recent announcement by Noguchi (*J. A. M. A.*, April 10, 1909) that human corpuscles may very well take the place of the corpuscles of the sheep in the test.

Although so short a period has elapsed since the epoch-making discovery of Wassermann, the field has attracted an army of workers. From all quarters comes confirmation of the great value and accuracy of the method, and, although more time will be necessary to verify many of the claims put forth by enthusiasts, enough is now known to warrant the belief that practically every case of syphilis will be earlier and more certainly diagnosed than hitherto by the adoption of this procedure.

That the serum reaction is specific for syphilis is assured. The failures are infinitesimal. Butler found that in from 90 to 95 per cent of all cases presenting syphilitic manifestations the reaction was specific. Latent cases show a specificity of from 50 to 60 per cent. The percentage of parasyphilitic cases runs from 70 to 80. The small proportion reported negatively may, in part, be possibly attributed to mistakes due to the difficult and complicated technique required for the test.

It would appear that the reaction is more positive in those cases not under treatment, suggesting the advisability in doubtful cases of suspending treatment temporarily. The value of a diagnostic method of so great accuracy can not be overestimated. To be able to state positively soon after the appearance of an initial lesion that syphilis is present gives us an opportunity to commence treatment earlier and avoids the necessity of delaying it until the appearance of secondary symptoms. Where the reaction is positive treatment may be instituted at once.

According to Marchildon (*St. Louis Med. Rev.*, Jan. 9, 1909), "in the presence of hard chancre, with a negative Wassermann reaction the chancre may be excised surgically and syphilis aborted. With a chancre present and serum reaction positive, then general infection has taken place and excision of chancre can be of no benefit."

The field of usefulness for the reaction is a large one. Not only will it be found valuable in all doubtful primary and secondary forms of the disease, but in the ofttimes vague tertiary stage must it prove of great value. In such conditions as brain syphilis, cerebrospinal syphilis, hemiplegia, tabes, epilepsy and neuritis, the advantage of a specific test in the diagnosis is obvious. While negatively the reaction has not proved itself infallible, it is sufficiently certain to be of use often in making a diagnosis by exclusion. In cases of pseudoluetic rheumatism or in pregnant women where there is a suspicion of syphilis, the test is important. It should be especially valuable in determining positively the existence of syphilophobiaism.

In Germany the life insurance companies require the test of all applicants for insurance, which would appear the part of wisdom in view of the report that 20 per cent of all male adults of Berlin have syphilis in some form. That the value of Wassermann's reaction is far-reaching is evidenced by the announcement of Lederman (*Med. Klinik,* Berlin, March 21, 1909), that he found it to be positive in sixteen children who inherited syphilis.

In those cases where the spirocheta pallida can be demonstrated the Wassermann reaction need only be used as a confirmatory procedure. Its great field of usefulness lies among that large class of cases where a serum test is the only available..—*Maryland Medical Journal.*

